# Assessment of treatment outcomes in patients with breast cancer who underwent mastectomy and breast reconstruction with sparing of the areola-nipple complex – single-center analysis

**DOI:** 10.3389/fonc.2026.1706468

**Published:** 2026-05-13

**Authors:** Wojciech Stachura, Magdalena Nowikiewicz, Marek Zdrenka, Maria Szymankiewicz, Joanna Stachura, Iwona Głowacka-Mrotek, Magdalena Tarkowska, Łukasz Szylberg, Wojciech Zegarski, Tomasz Nowikiewicz

**Affiliations:** 1 Department of Obstetrics, Pregnancy Pathology and Gynaecology, MSWiA Hospital, Bydgoszcz, Poland; 2 Department of Hepatobiliary and General Surgery, Dr. Antoni Jurasz University Hospital, Bydgoszcz, Poland; 3 Department of Tumor Pathology and Pathomorphology, Oncology Centre, Prof. Franciszek Łukaszczyk Memorial Hospital, Bydgoszcz, Poland; 4 Department of Microbiology, Oncology Centre, Prof. Franciszek Łukaszczyk Memorial Hospital, Bydgoszcz, Poland; 5 Department of Ophtalmology, Nicolaus Copernicus University in Toruń, Collegium Medicum in Bydgoszcz, Bydgoszcz, Poland; 6 Department of Rehabilitation, Nicolaus Copernicus University in Toruń, Collegium Medicum in Bydgoszcz, Bydgoszcz, Poland; 7 Department of Urology, Nicolaus Copernicus University in Toruń, Collegium Medicum in Bydgoszcz, Bydgoszcz, Poland; 8 Department of Obstetrics, Gynaecology and Oncology, Nicolaus Copernicus University in Toruń, Collegium Medicum in Bydgoszcz, Bydgoszcz, Poland; 9 Chair of Pathology, Dr. Jan Biziel University Hospital, Bydgoszcz, Poland; 10 Department of Surgical Oncology, Nicolaus Copernicus University in Toruń, Collegium Medicum in Bydgoszcz, Bydgoszcz, Poland; 11 Department of Clinical Breast Cancer and Reconstructive Surgery, Oncology Centre, Prof. Franciszek Łukaszczyk Memorial Hospital, Bydgoszcz, Poland

**Keywords:** breast cancer, Breast cancer surgery, breast reconstruction (BR), early and late treatment results, skin-sparing mastectomy and nipple-areola complex

## Abstract

**Purpose:**

Despite significant improvements in early detection of breast cancer, some patients still require mastectomy. One clinical problem currently being analyzed is the possibility of preserving the areola-nipple complex (NAC). The aim of this study was to evaluate the treatment results of breast cancer patients who underwent mastectomy with breast reconstruction with sparing of the NAC.

**Methods:**

335 patients who underwent mastectomy with breast reconstruction – with sparing (group I: 300 patients) or removal of NAC (group II: 35 patients), treated in the period 07.2014-06.2020. In the study groups, the length of overall survival (OS) and recurrence-free survival (RFS) were determined – up to 24 months after the end of treatment (short-term results) and up to 80 months (long-term results). The mean follow-up time of patients was 55.6 ± 19.7 months.

**Results:**

In 50 patients (14.9%), recurrence of neoplastic disease was observed (in group I: 14.7%, in group II: 17.1%; p=0.6972). It was most often a recurrence of cancer in the scar after mastectomy – in 25 patients (respectively: 7.7% vs 5.7%; p=0.6775) or distant metastases – in 16 patients (4.0% vs 11.4%; p=0.051). A total of 10 deaths were observed (6 – 2.0% vs 4 – 11.4%; p=0.0019). A total of 37 patients (10.7% vs 14.3%) required removal of their implant, mainly due to symptoms of infection – in 24 cases (7.3% vs 5.7%).

**Conclusion:**

Preserving the NAC is a safe therapeutic procedure in patients undergoing mastectomy with the option of reconstructive treatment. In both groups of patients, similar early and late treatment results were obtained.

## Introduction

Breast conserving treatment (BCT) has been a standard therapeutic option for patients with early invasive breast cancer for many years (1, 2). Its effectiveness and safety have been demonstrated in primary studies (3, 4). This has also been confirmed by the results of subsequent randomized clinical trials (5, 6).

However, despite significant improvements in early detection of breast cancer, some patients still require mastectomy. The recommended method of reducing the adverse effects of mastectomy is reconstruction of the breast during the same procedure (1, 2). Among the currently available technical solutions, the preferred method of breast reconstruction is nipple and skin-sparing mastectomy (NSSM), with prepectoral placement of the implant. The main reason for this trend is the pursuit of the most favorable aesthetic effect of the treatment while maintaining the oncological safety of the procedure (7–10).

In addition to the risk of a number of complications typical of reconstructive procedures, the NSSM procedure differs significantly from other types of mastectomy. One current clinical problem being analyzed in patients undergoing NSSM is the safety of preserving the nipple-areolar complex (NAC) (7–9). This primarily concerns the possibility of cancer recurrence. The main risk factor for early local recurrence is, admittedly, non-radical excision of the primary tumor (11). However, in the case of late recurrences, observed from a few to nineteen years after the end of treatment, cancer develops in the glandular tissue left within the skin envelope of the implant. Its presence may concern a significant percentage of NSSM procedures, reaching, according to some authors, even 50-100% of cases (12, 13). This is the result of the planned sparing of the retroareolar area, including the terminal sections of the nipple ducts. However, there is no consensus on the clinical consequences of preserving the NAC in patients undergoing mastectomy. One promising way to address this issue is the ongoing prospective EURECCA trial (14).

The aim of the study was to evaluate early and long-term treatment outcomes in breast cancer patients who underwent NAC-sparing mastectomy with breast reconstruction.

## Methods

### Group of patients

The study was conducted as a retrospective analysis. The Bioethics Committee of the Collegium Medicum UMK in Bydgoszcz approved it (KB 675/2018 of 30.10.2018). The study included 335 patients with breast cancer who underwent mastectomy with one-stage breast reconstruction using a silicone implant, or two-stage reconstruction using a tissue expander, treated in our center between 07.2014 and 06.2020. Of these, 300 patients underwent surgery with sparing of NAC (group I), while in the remaining 35 cases NAC was removed (group II – SSM – skin-sparing mastectomy). The reason for this disproportion was the lack of a larger number of patients operated on by us with removal of NAC. The average age of the patients was 45.9 years (range 25–69 years). The decision to leave the NAC in place in patients in group I was preceded by an intraoperative histopathological examination of a tissue sample taken from the nipple area. This examination was carried out routinely, regardless of the location of the primary tumour and its distance from the NAC. If the examination revealed the presence of tumour tissue, the NAC was removed.

All surgical procedures were performed by surgeons with many years of experience in the surgical treatment of malignant breast tumors. The choice of the type of implant used during the procedure – a silicone prosthesis (for one-stage reconstruction) or a tissue expander (two-stage reconstruction, requiring subsequent replacement of the implant with a final prosthesis) – was determined by the surgeon. This also applied to the site of implant placement (under the pectoralis major muscle, and subcutaneously, above the muscle). Neither variable was the subject of detailed statistical analysis.

The principles of patient qualification for surgical treatment, as well as for neoadjuvant/induction or adjuvant treatment (chemo-, radio-, immuno-, hormone therapy), were consistent with the current recommendations for the treatment of breast cancer (1, 2).

### Study end points

The primary objective of the study was to determine overall survival (OS) and recurrence-free survival (RFS) up to 24 months after treatment completion (short-term outcomes) and up to 80 months (long-term outcomes).

OS was defined as the time between surgery and patient death, regardless of the cause of death. RFS was defined as the time between surgery and first recurrence (or patient death). In the case of cancer recurrence, its type (local recurrence, axillary recurrence, distant metastases) and location were determined.

The secondary objectives of the study were to determine the effects of selected clinical variables on OS and RFS of the patients. The frequency of infectious complications, including the need to remove the implant used during the reconstructive procedure, was also determined.

Postoperative monitoring of patients was conducted until June 2022. The mean follow-up time was 55.6 ± 19.7 months (range: 24 to 90 months).

### Evaluated clinical data

In order to achieve the objective of the study, selected epidemiological and clinicopathological data of the patients were statistically analyzed (age, size of the primary lesion, histological grade, histological type and biological type of cancer, presence of metastatic lesions in the axillary lymph nodes, type of surgery, type of combined treatment). Full characteristics of the studied patients are presented in **Table 1**.

**Table 1:** Clinical characteristics of patients qualified for the study – univariate analysis.

Evaluated parameter	All patients	Group I (preserving NAC)	Group II (removal of NAC)	p
Patient age [range]	45.9 (25-69)	47.7 (25-69)	45.6 (27-66)	0.23
Histological type - DCIS - invasive NST - invasive lobular - invasive – other types	51/335 (15.2%) 236/335 (70.4%) 28/335(8.4%) 13/335 (3.9%)	47/300 (15.7%) 211/300(70.3%) 25/300 (8.3%) 12/300 (4%)	4/35 (11.4%) 25/35 (71.4%) 3/35 (8.6%) 1/35 (2.9%)	0.509 0.893 0.9616 0.7404
Histological malignancy grade - G1 - G2 - G3 - nd	18/335 (5.4%) 180/335 (53.7%) 94/335 (28.1% 42/335 (12.5%)	15/300 (5%) 160/300 (53.3%) 87/300 (29%) 38/300 (12.7%)	3/35 (8.6%) 20/35 (57.1%) 7/35 (20%) 4/35 (11.4%)	0.375 0.6688 0.2621 0.834
Tumor stage - cT1 cT1a cT1b cT1c - cT2 - cT3	133/335 (39.7%) 7/335 (2., 1%) 28/335 (8.4%) 95/335 (28.4%) 152/335 (45.4%) 49/335 (14.6%)	123/300 (41%) 6/300 (2%) 25/300 (8.3%) 89/300 (29.7%) 135/300 (45%) 41/300 (13.7%)	10/35 (28.6%) 1/35 (2.9%) 3/35 (8.6%) 6/35 (17.1%) 17/35 (48.6%) 8/35 (22.9%)	0.155 0.7373 0.9616 0.1198 0.688 0.1454
Axillary lymph nodes status - cN0 - cN1	301/335 (89.9%) 34/335 (10.1%)	272/300 (90.7%) 28/300 (9.3%)	29/35 (82.9%) 6/35 (17.1%)	0.1477 0.1477
Clinical stage - IA - IIA - IIB - IIIA	126/335 (37.6%) 140/335 (41.8%) 60/335 (17.9%) 8/335 (2.4%)	117/300 (39%) 126/300 (42%) 49/300 (16.3%) 7/300 (2.3%)	9/35 (25.7%) 14/35 (40%) 11/35 (31.4%) 1/35 (2.9%)	0.1247 0.82 0.0275 0.8477
Molecular type - luminal A - luminal B HER2(-) - luminal B HER2(+) - non-luminal HER2(+) - tripple negative - nd	56/335 (16.7%) 109/335(32.5%) 30/335 (9%) 29/335 (8.7%) 50/335 (14.9%) 2/335 (0.6%)	52/300 (17, 3%) 95/300 (31.7%) 27/300 (9%) 26/300 (8.7%) 45/300 (15%) 2/300 (0.7%)	4/35 (11.4%) 14/35 (40%) 3/35 (8.6%) 3/35 (8.6%) 5/35 (14.3%) 0/35 (0%)	0.3756 0, 3194 0.933 0.9849 0.9106 0.628
Tumor type - primary tumor - cancer reccurence	325/335 (97%) 10/335 (3%)	291/300(97%) 9/300 (3%)	34/35 (97.1%) 1/35 (2.9%)	0.9625 0.9625
Type of implant - expander - prosthesis	268/335 (80%) 67/335 (20%)	240/300 (80%) 60/300 (20%)	28/35 (80%) 7/35 (20%)	1 1
Place of implant insertion - subpectoral - prepectoral	306/335 (91.3%) 28/335 (8.4%)	273/300 (91%) 26/300 (8.7%)	33/35 (94.3%) 2/35 (5.7%)	0.513 0.5504
ALND	88/335(26.3%)	74/300 (24.7%)	14/35 (40%)	0.0511
CHTH - before surgery - after surgery - no	72/335 (21.5%) 139/335 (41, 5%) 124/335 (37%)	70/300 (23, 3%) 117/300 (39%) 113/300 (37.7%)	2/35 (5.7%) 22/35 (62.9%) 11/35 (31.4%)	0.0163 0.0067 0.4695
RTH - before surgery - after surgery - no	8/335 (2.4%) 61/335 (18.2%) 266/335 (79.4%)	7/300 (2.3%) 50/300 (16.7%) 243/300 (81%)	1/35 (2.9%) 11/35 (31, 4%) 23/35 (65.7%)	0.8477 0.0322 0.0343

The clinical data were collected prospectively (maintained with full anonymity of patients) from a database. They also came from hospital records of outpatient treatment and from telephone surveys. This allowed us to obtain complete information on treatment outcomes for 330 patients (98.5%) included in the study.

### Statistical analysis

The calculations were performed using PS IMAGO PRO 8.0 software (Predictive Solutions sp. z o.o.) and Statistica (TIBCO Software Inc. (2017) version 13.3).

Quantitative relationships between categorized variables (amounts or frequencies) of feature occurrence were assessed using the Chi2 test (Pearson or Wald H0 statistic, respectively).

Associations between OS and RFS and continuous, ranked, and qualitative variables were estimated using the Cox proportional hazards model. OS or RFS were entered into the model as complete observations.

To assess whether NAC retention is an independent prognostic factor, due to the small number of complete observations (preventing the construction of a reliable multivariate model), a series of bivariate analyses were performed using the Cox proportional hazards model.

In all analyses, two-sided tests were performed and a cut-off value of p<0.05 was used.

## Results

The analyzed groups of patients did not differ statistically significantly in terms of the distribution of factors describing the primary tumor and clinical advancement of the disease (except for stage IIB; p=0.0275).

In order to reconstruct the breast, a tissue expander was implanted in 268 patients (80%), and in the remaining 67 cases a definitive implant was used. In 306 (91.3%) patients, the implant was placed under the pectoralis major muscle, and in 29 (8.7%) subcutaneously, above the muscle. A total of 211 patients were qualified for systemic treatment, of which 72 patients for preoperative chemotherapy (in group I: 23.3%, in group II: 5.7%; p=0.0163) and 139 for postoperative chemotherapy (39% vs 62.9%; p=0.0067). 61 patients underwent adjuvant radiotherapy (16.7% vs 31.4%; p=0.0322).

Recurrence of cancer was found in 50 patients in total (14.9%; in group I: 14.7%, in group II: 17.1%; p=0.6972). It was most often a recurrence of cancer in the scar after mastectomy – in 25 patients (7.7% vs 5.7%), dissemination of the disease – in 16 patients (4.0% vs 11.4%), or axillary recurrence of cancer – in 9 patients (3.0% vs 0%). In seven cases, cancer recurrence affected two locations at the same time – **Table 2**.

**Table 2:** Treatment results of the analyzed group of patients – univariate analysis.

Evaluated parameter	All patients	Group I (preserving NAC)	Group II (removal of NAC)	p
Patient’s deaths	10/335 (3%)	6/300 (2%)	4/35 (11.4%)	0.0019
Cancer reccurence -postmastectomy scar -nipple -armpit - distant metastases	50/335 (14.9%) 25/335 (7.5%) 7/335(2.1%) 9/335 (2.7%) 16/335 (4.8%)	44/300 (14.7%) 23/300(7.7%) 7/300 (2.3%) 9/300 (3%) 12/300 (4%)	6/35 (17.1%) 2/35 (5.7%) 0/35 (0%) 0/35 (0%) 4/35 (11.4%)	0.6972 0.6775 0.361 0.299 0.051
Implant loss	37/335 (11%)	32/300 (10, 7%)	5/35 (14, 3%)	0.518
Reason for implant removal - infection - others	24/37 (64.9%) 13/37 (35.1%)	22/32(68.8%) 10/32 (31.3%)	2/5 (40%) 3/5 (60%)	0.2104 0.6975

A total of 10 deaths were reported (6 – 2.0% vs 4 – 11.4%; p=0.0019).

In group I, a higher chance of overall survival up to 24 months after reconstructive surgery was observed, compared to patients from the control group (p=0.0421). No difference was observed between patients with preserved or removed NAC (p=0.2016) in the chance of survival up to 24 months without disease symptoms – **Table 3**.

**Table 3:** Evaluation of short-term treatment results in the analyzed groups of patients.

Evaluated parameter	Removal of NAC	Odds ratio (95% Confidence Interval)	p
Overall survival	Yes	1 (ref.)	p=0.0421
	No	0.149 (0.024;0.935)	
Recurrence-free survival	Yes	1 (ref.)	p=0.2016
	No	0.469 (0.147;1.500)	

Next, the influence of selected clinical variables on the length of OS and RFS was assessed in both study groups. For this purpose, Cox proportional hazard analysis was performed in terms of OS and RFS as a result of the treatment, in the 80-month follow-up period. The results are presented collectively in **Table 4**.

**Table 4:** Evaluation of long-term treatment results in the analyzed groups of patients.

Evaluated parameter	Disease - Free Survival (DFS)	Overall Survival (OS)
	p-value	
Clinical stage		
- all group - I - II - III	0.635 0.579 0.643 **0.014**	**0.001** 0.193 **0.038** **0.046**
Histological type		
- DCIS - invasive NST	0.715 0.943	**0.017** 0.056
Histological malignancygrade		
- G1 - G2 - G3	0.054 0.457 0.172	**0.006** **0.031** 0.698
Molecular type		
- luminal A - non-luminal HER2(+) - tripple negative	0.574 0, 144 0.711	0.808 **0.007** 0.565
ALND		
- yes - no	0.855 0.494	0.721 **0.001**
Type of implant		
- expander - prosthesis	0.516 **0.008**	0.143 **<0.001**
Tumour size – pathological evaluation		
- pT1 - pT2-3	0.527 0.121	**0.012** 0.790
Axillary lymph nodes – pathological evaluation		
- pN0 - pN1-3	0.911 0.992	**0.003** 0.566
CHTH		
- before surgery - after surgery - no	0.569 0.842 0.486	0.792 **0.044** **<0.001**
RTH		
- before surgery - after surgery - no	0.705 0.731 0.367	0.705 0.496 **<0.001**

A bivariate Cox proportional hazard analysis was also performed in terms of DFS and OS length, between NAC retention and age, BMI, tumor stage and differentiation, axillary lymphadenectomy, CHTH and RTH use, implant type, tumor size, and the presence of breast cancer metastases in the axillary lymph nodes. However, no effect of any of the above factors on DFS length was observed (p>0.05). However, for each of the above factors, a statistically significant effect of NAC retention on OS length was confirmed (p<0.05).

A total of 37 patients required removal of their implant (10.7% vs 14.3%, respectively; p=0.518). The main cause of this situation was wound infection that was not amenable to conservative treatment (targeted antibiotic therapy) – in 24 cases (68.8% vs 40.0%), and in the remaining cases it was local recurrence of cancer or implant damage.

## Discussion

Mastectomy is a surgical procedure whose original goal was to remove all tissues of the mammary gland. Since the introduction of this procedure, it has undergone a significant evolution in terms of gradually limiting the scope of removed structures. This resulted in reduced surgical trauma, improved aesthetic effect obtained after treatment, and it did not worsen the long-term results of anticancer therapy (7–9). The proposal of the possibility of preserving the NAC started a discussion on the clinical consequences of conserving the nipple in operated patients. Opponents of this option noted the potential risk of breast cancer recurrence within the preserved terminal sections of the milk ducts.

As mentioned in the introduction to this paper, the lack of removal of glandular breast tissue concerns mainly NSSM procedures, and to a lesser extent other types of mastectomies (12, 13). According to the results of the studies by Giannotti et al., it is least frequent after simple mastectomy (in 2.8% of patients), several times more common in the case of skin-sparing mastectomy (SSM – 13.2%), reaching 51% after NSSM (15). Similar data were also presented by the authors of other studies (12, 13, 16). However, according to Rocco et al. (17) and Zaborowski et al. (18), such a situation does not increase the risk of local cancer recurrence, and the NSSM procedure is an oncologically safe procedure.

Different conclusions were presented by Deutschmann et al. (19). In a study based on a nearly 5-year observation period of 105 patients who underwent therapeutic mastectomy, local recurrence of cancer occurred in 17 (16.2%) operated breasts. According to Skjerven et al., breast cancer recurrence occurs significantly more often after skin-sparing mastectomy compared to classic simple mastectomy (in a 5-year observation: 3.9% vs. 0.9%, in a 10-year observation: 6.2% vs. 0.9%) (20). Breast cancer can also be diagnosed in BRCA1/2 gene mutation carriers who have previously undergone surgery to reduce the risk of developing this malignant tumor. This situation may concern up to 37.5% of patients who underwent a prophylactic mastectomy (21).

In the clinical material we analyzed, a small number of relapses of neoplastic disease were found (in 14.9% of patients). A large group of patients (335 patients) was included in the study. The compared groups did not differ statistically significantly in terms of the distribution of most of the assessed epidemiological and clinicopathological factors. Both short and long-term treatment outcomes were directly dependent on the type of surgery. In the case of sparing NAC, breast cancer relapse occurred in 14.7% of patients (vs. 17.1% in the control group, p=0.6972). A lower number of deaths was also observed (2.0% vs. 11.4%; p=0.0019). However, cancer relapse in the mastectomy scar was slightly more frequent in this group of patients (7.7% vs. 5.7%, respectively; p=0.6775). Similar results were obtained by Yamashita et al. Regardless of NAC preservation or removal, they found a comparable frequency of regional breast cancer recurrences in the analyzed patients (5.8% vs. 6.0%). No statistically significant differences were observed in the case of OS and DFS (22). In the study by Parvez et al., local breast cancer recurrence occurred in 4.6% of 175 analyzed patients (23). In the case of dissemination of the disease, according to Margenthaler et al., it may concern about 1% of patients undergoing NSSM or SSM (24).

The more favorable treatment results noted in our study in patients with preserved NAC (especially the long-term ones – both in terms of OS and DFS) are not clinical evidence of the existence of a possible protective effect of sparing these structures. However, they allowed us to obtain convincing evidence of the complete safety of this surgical procedure. Therefore, attention to the final aesthetic effect of the procedure performed for oncological indications does not have to be in conflict with the primary goal of striving for permanent recovery of patients, especially in strictly defined clinical situations.

Similar conclusions were presented by Fu et al. (25). The analysis of treatment results of 5, 765 patients undergoing NSSM and 134, 528 patients undergoing radical mastectomy without sparing the skin of the mammary gland did not show any differences in the OS period between both groups of patients.

In the clinical material we analyzed, differences in the frequency of using preoperative systemic treatment are noticeable. In the case of group I, this percentage was 23.3% and was statistically significantly higher than in the control group (5.7%; p=0.0163). The reverse relationship was observed in the postoperative period (39% vs. 62.9%; p=0.0067, respectively). This was the result of changes in the rules for qualifying breast cancer patients for neoadjuvant (or induction) therapy observed in recent years (1, 2). According to the current treatment standard, in the case of diagnosed biological types of cancer with an unfavorable prognosis (triple-negative cancer, HER2-positive cancers), despite the resectability of neoplastic lesions, patients require systemic treatment first, and then surgical treatment. In turn, the more frequent use of adjuvant RTH in group II was the result of a higher percentage of primary tumors exceeding 5 cm in size and a more frequent presence of cancer metastases in the axillary lymph nodes.

The study also assessed the risk of infectious complications, especially in the group of patients with NAC sparing. As is well known, leaving the nipple during reconstructive surgery of the breast is a factor verified in many studies that increases the frequency of surgical site infection and cases of the necessity to remove the implant (22–27). NAC sparing may also cause ischemia of the nipple, and consequently the appearance of necrotic lesions. According to the results of the study by Kato et al., the described sequence of events may concern up to 4.79% of NAC sparing procedures, especially in patients with other coexisting risk factors for infection (26). According to Karian et al. (27) and Jensen et al. (28), the percentage of cases of necrotic lesions of the nipple is even higher, concerning 7-17% of operated patients.

The clinical material we analyzed also showed the existence of such a relationship. The overall rate of surgical site infection was 7.2% (24 patients). In group I, wound infection occurred in 7.3% of patients, in the comparison group – in 5.7% of patients (p=0.2104). These results are therefore comparable to those obtained in the above-mentioned studies. They also confirm the possibility of conserving NAC without a simultaneous significant increase in the frequency of infectious complications.

The presented study has several significant limitations. The first is the retrospective method of recruitment and assessment of the analyzed group of patients. Second, its value would have been increased by the use of clinical material also from other oncological centers and the extension of the follow-up period of patients. Moreover, the study did not assess the aesthetic results of surgical treatment. This was due to several reasons: a significant number of patients underwent another surgical procedure during the observation period (replacement of the expander used during the initial surgery with a definitive implant - this two-stage procedure concerned the majority of the analyzed patients - 268, i.e. 80.0%), the retrospective nature of the study, and the limited interest of patients in participating in an additional anthropometric examination. Thirdly, the lack of an in-depth analysis of the infection identified in the study, and in particular the lack of an assessment of the impact of other risk factors for implant-related postoperative infections (i.e. the number and type of surgical procedures, the techniques used, the duration of procedures, and postoperative protocols) may influence the infection results obtained.

However, notwithstanding these remarks, the conducted analysis aptly fits into the ongoing discussion on all clinical consequences of maintaining NAC in patients requiring mastectomy. Additionally, the group of patients analyzed was representative of the population of a country with a high increase in the number of new breast cancer cases (by 2.42% per year) as well as high mortality due to this cause (3.27%) (29, 30).

## Conclusions

NAC preservation is a safe therapeutic procedure in patients undergoing mastectomy with the option of reconstructive treatment. In both compared groups of patients, similar short and long-term treatment results were achieved. Although leaving the nipple in place is an independent risk factor for postoperative site infection, NAC preservation did not increase the frequency of infectious complications accompanying reconstructive procedures using artificial implants.

## Data Availability

The original contributions presented in the study are included in the article/supplementary material. Further inquiries can be directed to the corresponding authors.
